# Efficacy and safety of nemolizumab in prurigo nodularis: a systematic review and meta-analysis of randomized controlled trials^؆^

**DOI:** 10.1016/j.abd.2025.501210

**Published:** 2025-10-18

**Authors:** Ana Carolina Putini Vieira, Maria Antônia Costa Cruz Akabane, Gianna Carolinne Graff Caletti, Kélen Klein Heffel, Amanda Tauana Oliveira e Silva

**Affiliations:** a Department of Medicine, Universidade de Santo Amaro, São Paulo, SP, Brazil; b Department of Medicine, Universidade Federal de Juiz de Fora, Juiz de Fora, MG, Brazil; c Department of Medicine, Universidade de Passo Fundo, Passo Fundo, RS, Brazil; d Department of Dermatology, Santa Casa de Misericórdia de Porto Alegre, Porto Alegre, RS, Brazil; e Department of Dermatology, Universidade Federal do Piauí, Teresina, PI, Brazil

**Keywords:** Immunomodulation, Monoclonal antibody, Prurigo, Pruritus

## Abstract

**Background:**

Prurigo nodularis (PN) is a chronic, debilitating inflammatory skin disorder characterized by persistent itching, firm pruritic nodular lesions, and evidence of frequent scratching, like excoriation, and lichenification. Nemolizumab, a monoclonal antibody targeting IL-31, has shown significant improvements in skin lesions, itching, and sleep disturbances by reducing type 2 immune responses in PN.

**Objectives:**

Evaluate the efficacy and safety of nemolizumab in treating PN by systematically analyzing randomized controlled trials (RCTs). Methods: The authors conducted a systematic review and meta-analysis by searching Pubmed, Embase, Cochrane Central and Scopus for RCTs comparing nemolizumab to placebo for PN. Statistical analysis used*R* Studio 4.3.2.

**Results:**

Three trials, involving 630 patients, were included. Nemolizumab significantly reduced pruritus at week-4 (MD = −32.04; 95% CI: −38.47, −25.62) and at week-16 (MD = −347.34; 95% CI: −1039.71, 345.04). Investigator’s Global Assessment (IGA) success favored Nemolizumab at week-16 (RR = 3.50; 95% CI: 2.18, 5.63). No significant differences were observed in adverse events (RR = 1.10; 95% CI: 0.97, 1.25) or serious adverse events (RR = 0.77; 95% CI: 0.43, 1.39). Nemolizumab also significantly reduced neurodermatitis.

**Study limitations:**

Limitations include variability in treatment duration, small sample sizes, and the lack of direct comparisons with other biologics.

**Conclusion:**

Our meta-analysis shows that Nemolizumab significantly improves pruritus, IGA success rates, and PAS > 75% in treating patients with moderate to severe PN. Its safety profile is favorable, with no significant differences in adverse events compared to placebo. These findings support Nemolizumab as a viable treatment for moderate to severe PN.

## Introduction

Prurigo nodularis (PN) is a chronic, debilitating inflammatory skin disorder characterized by persistent itching, firm pruritic nodular lesions, and evidence of frequent scratching, like excoriation and lichenification. It is considered a relatively uncommon illness, impacting 72 out of every 100,000 people in the United States, and primarily affects middle-aged to older adults, women, and individuals of African descent.^1^, ^2^ The condition can also affect patients’ mental and emotional health, being strongly linked to mental health issues, including depression, anxiety, and inclinations toward self-harm or suicidal thoughts.^3^

PN pathogenesis has been linked to an intense inflammatory response in the skin, with dense dermal infiltrates and cell release mediators such as Interleukin (IL)-13, other cytokines, and neuropeptides, which contribute to inflammation and intense pruritus. Neural dysregulation is also evident, with increased dermal innervation and elevated Nerve Growth Factor Receptor (NGFR) and PGP-9.5 density. At the same time, the epidermis lacks NGFR-positive fibers and has reduced PGP-9.5 fibers, likely due to repetitive scratching. Dysregulated neuropeptides, including elevated levels of substance P and calcitonin Gene-Related Peptide (CGRP), further contribute to PN pathogenesis. Substance P activates neurokinin-1 receptors, while CGRP drives neurogenic inflammation, regulates eosinophils and mast cells, disrupts opioid receptor expression, and intensifies pruritus.^4^, ^5^

Therapies for PN are limited, with dupilumab and the recently approved nemolizumab being the only systemic treatments approved by the US Food and Drug Administration (FDA).^6^, ^7^ Other available pharmacological interventions are off-label, although there is notable variation in treatment regimens, therapeutic aims, and safety/effectiveness. Interleukin-31, a cytokine that elicits itch sensation, is significantly increased in PN patients in comparison with healthy individuals; therefore, blocking interleukin-31 presents a highly promising therapeutic strategy for addressing the disease.^5^, ^7^

Nemolizumab, a humanized monoclonal antibody, disrupts IL-31 by binding to the IL-31 receptor α subunit and blocking receptor activation signaling. This reduces type 2 immune responses in PN, easing the disease’s underlying inflammation and symptoms.^8^ Current clinical studies have shown significant improvements in skin lesions, itching, and sleep disturbances with Nemolizumab treatment.^1^, ^9^, ^10^

A previous network meta-analysis has assessed the efficacy of treatment choices for PN, including nemolizumab, although it incorporated only one randomized controlled trial (RCT) assessing nemolizumab.^11^ Nilforoushzadeh et al. conducted a systematic review on interleukin-31 inhibitors for PN treatment, yet they did not perform a meta-analysis to provide a more precise estimate of effect size and included a broader range of study designs, such as cohort studies.^12^ While these studies have provided valuable insights into the efficacy and safety of Nemolizumab, there is a need for a comprehensive synthesis of the latest available data to better understand the benefits and risks of this agent in patients with PN. Our meta-analysis fills this gap by including three high-quality RCTs, enabling a more precise evaluation of the efficacy and safety of nemolizumab in prurigo nodularis. By focusing solely on RCTs, the authors provide a clearer, evidence-based understanding of the treatment's benefits and risks, crucial for guiding clinical practice and future research.

Therefore, this meta-analysis aims to systematically review and synthesize the available evidence on the efficacy and safety of Nemolizumab in patients with PN.

## Methods

This systematic review and meta-analysis were conducted in accordance with the protocols established by the Cochrane Collaboration and adhered to the guidelines outlined in the Preferred Reporting Items for Systematic Reviews and Meta-Analyses (PRISMA). The study protocol was preregistered in the International Prospective Register of Systematic Reviews (PROSPERO) under the identification number CRD42024626990.

### Search strategy and Data extraction

The authors systematically searched databases, including PubMed, Embase, Scopus, and Cochrane Central Register of Controlled Trials, from inception to December 12, 2024, following the PRISMA Statement guidelines and the Cochrane Collaboration Handbook for Systematic Reviews of Interventions. The following search terms were employed: “nemolizumab”, “prurigo nodularis”, and “prurigo”. The complete search strategy is available in the Supplementary Material.

Two authors (ACPV and KKH) independently extracted data on study characteristics, interventions, and outcomes of interest. Any discrepancies were addressed through consensus among the authors.

Outcomes evaluated included: Percent change from baseline in Peak Pruritus Numerical Rating Scale (PP-NRS) at 4-, 12- and 16-weeks, Investigator’s Global Assessment (IGA) success at 12-weeks, Prurigo Activity Score (PAS) >75% healed pruriginous lesions at 12- and 16-weeks, adverse events (AEs), serious adverse events (SAEs), any adverse events leading to discontinuation, alopecia, cardiac disorders, contact dermatitis, dry skin, eczema, peripheral edema, gastrointestinal disorders, infections and infestations, injection related reaction, musculoskeletal and connective tissue disorders, nervous system disorders, neurodermatitis, rash, rosacea, urticaria.

### Eligibility criteria and study selection

Two reviewers (ACPV and GCGC) independently screened the articles for inclusion, with any discrepancies resolved by consensus. Inclusion in this meta-analysis was restricted to studies that met all the following eligibility criteria: 1) RCTs; 2) Comparing Nemolizumab therapy with placebo; 3) Reporting at least one clinical outcome such as PP-PRS or adverse events. Exclusion criteria included: 1) No control group; 2) Not reporting outcomes of interest; 3) Non-randomized designs; 4) Studies only available in abstract form; 5) Post hoc analysis; 6) Uncompleted clinical trials; 7) Studies involving concomitant corticosteroid use.

### Quality assessment

Two authors (ACPV and GCGC) assessed the quality of the included studies. As suggested by Cochrane, risk of bias was assessed using the Cochrane Risk-of-Bias tool for randomized trials (RoB-2).

### Statistical analysis

This systematic review and meta-analysis were performed and reported following the Cochrane Collaboration Handbook for Systematic Review of Interventions and PRISMA Statement guidelines. Standardized Mean Differences (SMD) with 95% Confidence Intervals were used to compare continuous outcomes. The authors assessed heterogeneity with I² statistics and the Cochran *Q* test; p-values < 0.10 and I² > 25% were considered significant for heterogeneity. Statistical analysis was performed using R Studio 4.3.2.

## Results

### Study selection and baseline characteristics

**Fig. 1:**
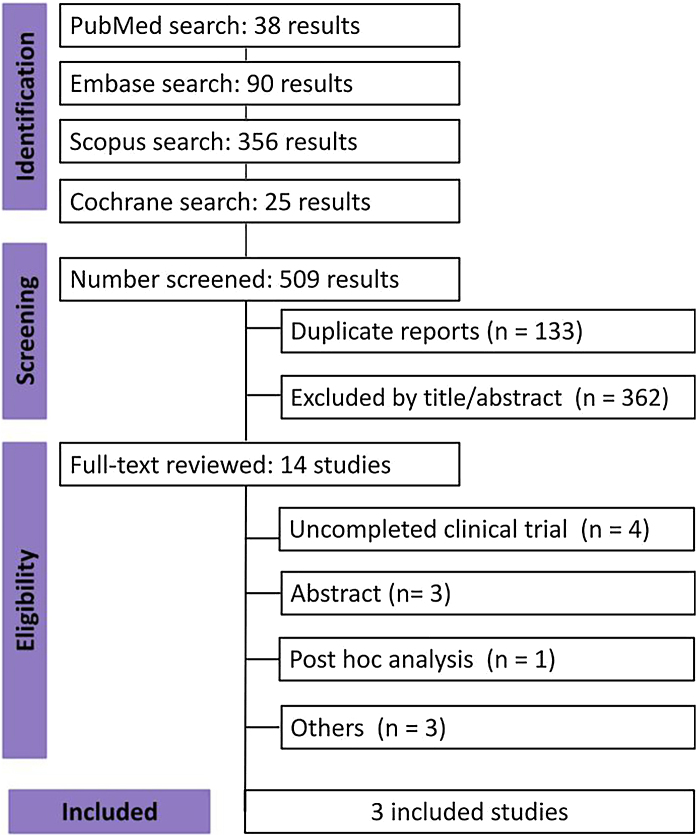
PRISMA search flow diagram.

As shown in Fig. 1, the initial search identified 509 results. After eliminating duplicate records and ineligible studies based on title and abstract, 14 studies remained for full-text review based on the inclusion criteria. Of these, three studies were included in this systematic review and meta-analysis, comprising 630 patients. Among these, 407 (65%) patients received Nemolizumab, while 223 (35%) were given a placebo. Follow-up periods ranged from 16 to 32 weeks. The mean age of participants varied from 50.8 to 59.7 years, with a mean weight ranging from 79.7 to 87.1 kg. Study and patient characteristics are summarized in Table 1.^1^, ^9^, ^10^

**Table 1:** Baseline characteristics of included studies.

Study, Year	Eligibility Criteria	Treatment Duration	Patients (n) NEMO/PB	Age^a^ (years) NEMO/PB	Female (%) NEMO/PB	Weight^a^ (kg) NEMO/PB	Weekly average PP-NRS score^a^ NEMO/PB	DLQI^a^ NEMO/PB
Ständer et al. 2024 (Olympia 1) ^10^	Adults with PN ≥ 6 months, severe pruritus (PP-NRS ≥7 in the week prior to baseline), ≥20 bilaterally distributed nodules (upper/lower limbs or trunk), and IGA ≥3.	24 weeks	190/96	57.5/57.6	57.9/58.3	87.1/80.8	8.5 /8.4	17.1/16.9
Kwatra et al. 2023 (Olympia 2) ^1^	Adults ≥18 with PN ≥ 6 months, severe pruritus (PP-NRS ≥7 in the week prior to baseline), ≥20 bilaterally distributed nodules (arms, legs, or trunk), and IGA score of 3 or 4.	16 weeks	183/91	53.7/50.8	61.7/60.4	79.7/80.8	8.5/8.4	16.5/17.1
Ständer et al. 2020 ^9^	Adults with PN and severe pruritus (mean score ≥7 for ≥6 months), ≥20 bilateral nodules on the upper limbs, with or without trunk/lower limb.	12 weeks	34/36	59.7/52.4	55.8/61.1	81.6/80.3	8.4/8.4	16.9/15.8

PN, Prurigo Nodularis; PP-NRS, Peak Pruritus Numerical Rating Scale (0 = none, 10 = worst itch); IGA, Investigator’s Global Assessment (0‒4; higher scores = more severe prurigo nodularis); NEMO, Nemoluzimab; PB, Placebo; DLQI, Dermatology Life Quality Index (0‒30; higher scores = more quality of life impairment).

a Mean or median.

### Dose and duration of nemolizumab therapy

In Ständer et al. 2024, the trial included a screening period of 1- to 4-weeks, a 24-week treatment phase, and an 8-week follow-up. Participants were randomized to receive subcutaneous injections of nemolizumab monotherapy or matching placebo every 4-weeks for 24-weeks. Patients weighing < 90 kg received an initial dose of nemolizumab, 60 mg, followed by 30 mg every 4-weeks, while those weighing ≥ 90 kg received 60 mg of nemolizumab every 4-weeks.

In Kwatra et al., the study featured a screening period of 1- to 4-weeks, a 16-week treatment phase, and an 8-week follow-up. Patients were randomized to receive subcutaneous injections of nemolizumab or matching placebo, with those weighing <90 kg receiving an initial dose of 60 mg (administered as two 30 mg injections) followed by 30 mg every 4-weeks, and those weighing ≥ 90 kg receiving 60 mg (two 30 mg injections) every 4-weeks.

The Ständer et al. 2020 study featured a 12-week treatment phase and follow-up visits at weeks-16 and -18. Patients were randomized to receive nemolizumab at a dose of 0.5 mg per kilogram of body weight or placebo, with subcutaneous injections administered at baseline, week-4, and week-8. Rescue therapy for pruritus was allowed from day-29 onward, with topical therapies permitted while continuing nemolizumab or placebo, and systemic therapies requiring discontinuation of the study drug.

### Pooled analysis of all studies

**Fig. 2:**
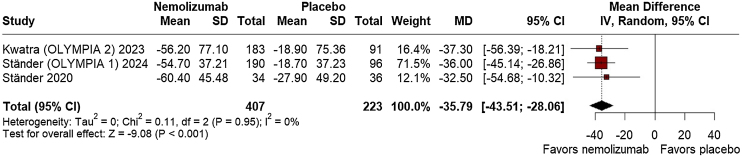
Percent change from baseline in peak pruritus numerical rating scale at 16 weeks.

**Fig. 3:**
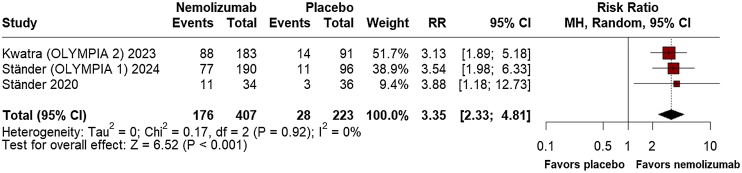
Investigator's Global Assessment (IGA) success rates at 12 weeks.

**Fig. 4:**
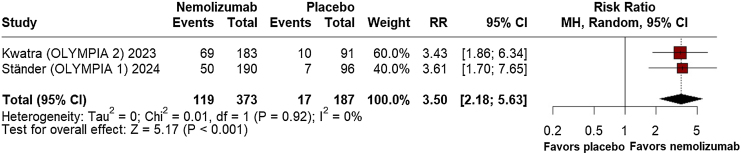
Investigator's Global Assessment (IGA) success rates at 16 weeks.

Regarding efficacy outcomes, nemolizumab demonstrated a significant percent change from baseline in the Peak Pruritus Numerical Rating Scale Least Squares (LS) Mean at week-4 (MD = −32.04; 95% CI: −38.47, −25.62; p ≤ 0.001; I^2^ = 0%; Supplement Fig. S1), favoring the intervention. At week-12, however, no significant difference was observed between the groups (MD = −347.34; 95% CI: −1039.71, 345.04; p = 0.325; I^2^ = 0%; Supplement Fig. S2). By week-16, nemolizumab again showed considerable improvement in pruritus reduction (MD = −35.79; 95% CI: −43.51, −28.06; p ≤ 0.001; I^2^ = 0%; Fig. 2). Investigator's Global Assessment (IGA) success rates consistently favored nemolizumab at week-12 (RR = 3.35; 95% CI: 2.33, 4.81; p ≤ 0.001; I^2^ = 0%; Fig. 3), with a significant relative increase at week-16 (RR = 3.50; 95% CI: 2.18, 5.63; p ≤ 0.001; I^2^ = 0%; Fig. 4).

**Fig. 5:**
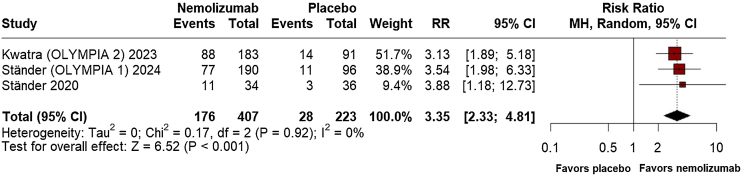
Prurigo Activity Score (PAS) >75% healed pruriginous lesions at 12 weeks.

**Fig. 6:**
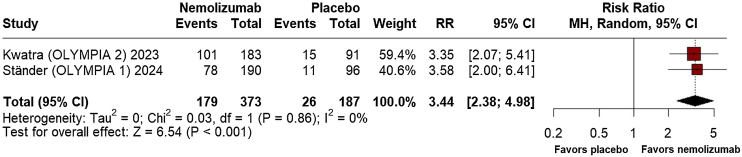
Prurigo Activity Score (PAS) >75% healed pruriginous lesions at 16 weeks.

In terms of the proportion of patients achieving PAS > 75, the results at week-12 favored nemolizumab (RR = 3.35; 95% CI: 2.33, 4.81; p ≤ 0.001; I^2^ = 0%; Fig. 5), while at week-16, nemolizumab demonstrated superior outcomes (RR = 3.44; 95% CI: 2.38, 4.98; p ≤ 0.001; I^2^ = 0%; Fig. 6).

**Fig. 7:**
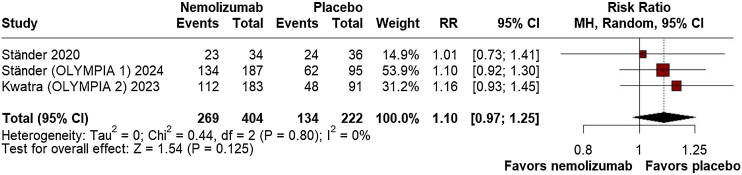
Adverse Events (AEs).

**Fig. 8:**
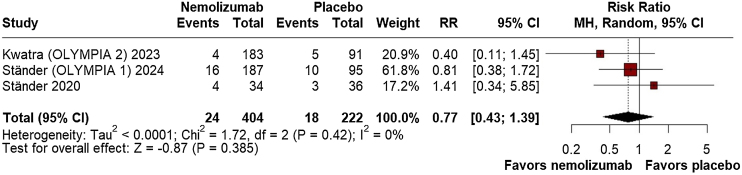
Serious Adverse Events (SAEs).

For safety outcomes, there was no significant difference between nemolizumab and placebo in the overall incidence of AEs (RR = 1.10; 95% CI: 0.97, 1.25; p = 0.125; I^2^ = 0%; Fig. 7) or SAEs (RR = 0.77; 95% CI: 0.43, 1.39; p = 0.385; I^2^ = 0%; Fig. 8). The proportion of patients discontinuing the study due to AEs was also not significantly different (RR = 1.09; 95% CI: 0.46, 2.54; p = 0.849; I^2^ = 0%; Supplement Fig. S3), and injection-related reactions were infrequent and not significantly different between groups (RR = 2.31; 95% CI: 0.38, 14.02; p = 0.364; I^2^ = 0%; Supplement Fig. S4).

Specific adverse events, such as peripheral edema (RR = 1.54; 95% CI: 0.40, 6.00; p = 0.530; I^2^ = 0%; Supplement Fig. S5), urticaria (RR = 1.94; 95% CI: 0.31, 12.19; p = 0.479; I^2^ = 0%; Supplement Fig. S6), contact dermatitis (RR = 3.62; 95% CI: 0.64, 20.35; p = 0.145; I^2^ = 0%; Supplement Fig. S7), and other dermatological conditions including eczema, alopecia, rash, rosacea, and dry skin, were not significantly different between groups (Supplement Figs. S8‒S12). Interestingly, nemolizumab showed a significant reduction in neurodermatitis compared to placebo (RR = 0.50; 95% CI: 0.32, 0.80; p = 0.004; I^2^ = 11%; Supplement Fig. S13).

Other system-related AE, such as cardiac disorders (RR = 0.56; 95% CI: 0.14, 2.20; p = 0.411; I^2^ = 0%; Supplement Fig. S14), gastrointestinal disorders (RR = 0.89; 95% CI: 0.48, 1.65; p = 0.707; I^2^ = 19%; Supplement Fig. S15), and skin and subcutaneous tissue disorders (RR = 1.03; 95% CI: 0.79, 1.35; p = 0.89; I^2^ = 0%; Supplement Fig. S16), were not significantly different. Infections and infestations (RR = 0.98; 95% CI: 0.66, 1.44; p = 0.899; I^2^ = 0%; Supplement Fig. S17), musculoskeletal and connective-tissue disorders (RR = 1.48; 95% CI: 0.89, 2.46; p = 0.132; I^2^ = 0%; Supplement Fig. S18), nervous system disorders (RR = 1.10; 95% CI: 0.65, 1.84; p = 0.730; I^2^ = 0%; Supplement Fig. S19), and respiratory, thoracic and mediastinal disorders (RR = 0.93; 95% CI: 0.29, 3.01; p = 0.3839; I^2^ = 0%; Supplement Fig. S20) also showed no significant differences.

These findings provide a comprehensive overview of the efficacy and safety profiles of Nemolizumab, highlighting its potential benefits in reducing pruritus and improving IGA success rates. It also has a favorable safety profile with no major safety concerns compared to the placebo.

### Quality assessment

**Fig. 9:**
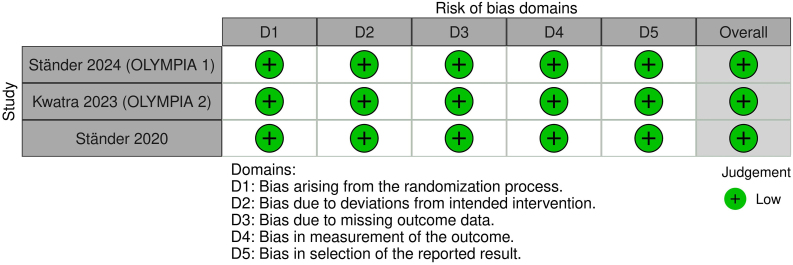
Risk of Bias assessment of included studies.

The risk of bias assessment for each RCT included in this meta-analysis was conducted using the RoB-2 tool, and all studies were deemed as low risk of bias (Fig. 9).

Domain 1 (Randomization Process) demonstrated a low risk of bias across all studies. The allocation sequence was reported as random, baseline differences between intervention groups did not indicate issues with randomization, and the allocation sequence was adequately concealed until participants were enrolled and assigned to interventions.

Domain 2 (Deviations from Intended Interventions) showed that participants were likely unaware of their assigned interventions during the trials, rated as “Probably No” for all studies. Carers and intervention providers were also unaware of participants’ assigned interventions, rated as “No”. Additionally, all studies employed an appropriate analysis to estimate the effect of assignment to intervention, rated as “Yes”.

Domain 3 (Missing Outcome Data) was assessed as low risk, as all studies reported data for all or nearly all randomized participants.

In Domain 4 (Measurement of Outcomes), the methods used to measure outcomes were deemed appropriate, with no evidence of differences in measurement or ascertainment between intervention groups. Outcome assessors were likely unaware of the intervention received by participants, rated as “Probably No” for all studies.

Finally, Domain 5 (Selection of the Reported Result) revealed that the data were analyzed according to a pre-specified plan finalized before unblinded outcome data were available, rated as “Yes” for all studies. There was no evidence of selective reporting from multiple eligible outcome measurements or analyses, rated as “No” for all studies.

## Discussion

This systematic review and meta-analysis of three RCTs, including 630 patients, compared nemolizumab with placebo for prurigo nodularis. The main findings were: 1) Nemolizumab significantly improved pruritus, with greater reductions in the Peak Pruritus Numerical Rating Scale at weeks-4, -12, and -16, higher IGA success rates at week-12, and superior outcomes in PAS > 75% healed lesions at week-16; 2) While no significant differences were observed in the incidence of overall or serious adverse events between groups, nemolizumab significantly reduced neurodermatitis compared to placebo. Other safety outcomes showed no substantial differences, reinforcing the favorable safety profile of nemolizumab.

Nemolizumab is a monoclonal antibody that targets the Interleukin-31 Receptor A (IL-31RA).^13^ IL-31, a cytokine associated with itching and the pathophysiology of prurigo nodularis, is crucial in T-helper 2 skin inflammation by promoting the production of IL-4 and IL-13. Furthermore, IL-31 triggers itch sensation in the peripheral nervous system by interacting with sensory nerve fibers. Through inhibiting IL-31, nemolizumab interrupts the neuroimmune-driven itch-scratch cycle, suppresses inflammation, enhances epithelial function, and strengthens the skin barrier. Nemolizumab has also been seen to suppress keratinocyte proliferation post-treatment.^8^, ^14^

The management of prurigo PN is determined by disease severity. Mild PN is typically managed with topical treatments such as corticosteroids, calcineurin inhibitors, and antihistamines. However, moderate-to-severe PN often requires systemic interventions due to its significant impact on quality of life and the often inadequate response to topical therapies alone.^15^ In the trials included in the present study, only patients with moderate-to-severe PN were enrolled, highlighting that nemolizumab is most effective for individuals with this level of severity.

Nemolizumab (Nemluvio®) has been approved by the US Food and Drug Administration (FDA) for subcutaneous injection to treat adults with prurigo nodularis.^6^ It is recommended for subcutaneous dosage only. For adults weighing less than 90 kg, the initial dose is 60 mg (administered as two 30 mg injections), followed by 30 mg every 4-weeks, and for adults weighing 90 kg or more, the initial dose is 60 mg (administered as two 30 mg injections), followed by 60 mg every 4-weeks.^16^

This medication is contraindicated in patients who have known hypersensitivity to nemolizumab-ilto or any of the excipients. Data on its use during pregnancy or breastfeeding is insufficient. However, it was detected in the breast milk of monkeys following subcutaneous doses up to 25 mg/kg once every two weeks during organogenesis to parturition. Additionally, the safety and effectiveness of nemolizumab (Nemluvio®) have not been established in pediatric patients, being only approved for adults over 18, with limited data on individuals aged 65 or older.^16^, ^17^

In this meta-analysis, superior efficacy outcomes were observed in the nemolizumab group compared to placebo at week-16, including a significant reduction in pruritus and higher IGA success rates. Statistically significant differences were evident for pruritus reduction as early as week-4, with nemolizumab showing considerable improvement. By week-12, patients receiving placebo had achieved a greater PAS > 75 score, but by week-16, success rates favored nemolizumab.

The present findings support the use of nemolizumab as an effective and safe treatment option for prurigo nodularis, aligning with previous research on biologics targeting the IL-31 pathway. For instance, Sofen et al. demonstrated similar efficacy with vixarelimab, another IL-31 inhibitor.^18^ A network meta-analysis comparing different treatments for PN to placebo, including vixarelimab, dupilumab, serlopitant, nalbuphine, and nemolizumab, showed that nemolizumab has the potential to improve pruritus and skin lesions in PN patients.^11^ While dupilumab is considered an excellent choice for treating PN, nemolizumab appears to have better results in reducing the extent and severity of skin lesions. Furthermore, Brooks et al., a review on nemolizumab, showed that when compared to dupilumab, nemolizumab performs similarly, further emphasizing its potential as a key treatment option.^14^

Moreover, the analysis of adverse outcomes revealed no significant differences in the overall incidence of AEs or SAEs, indicating favorable safety data for nemolizumab. Although peripheral edema, worsening of eczema were observed in the Olympia 1, Olympia 2, and Stander et al. 2020 trials, as well as in other studies investigating atopic dermatitis, the meta-analysis showed no significant difference between groups.^1^, ^9^, ^10^, ^19^, ^20^, ^21^, ^22^

In the present study, nemolizumab showed a significant reduction in neurodermatitis compared to placebo. This may be explained by the common features between neurodermatitis, also known as lichen simplex chronicus, and PN. Both conditions are characterized by epidermal hyperplasia, orthokeratosis, and hypergranulosis, resulting from repetitive scratching and rubbing of the skin.^23^ Nemolizumab, by targeting specific pathways of pruritus and inflammatory responses, breaks this itch-scratch cycle, reducing pruritus.^13^

This meta-analysis has several strengths, including the rigorous design of the included trials, which were randomized, placebo-controlled, and double-blinded, and consistent findings that enhance the reliability of the results. However, there are some limitations. One is the variability in treatment duration, as Ständer et al. 2020 report outcomes only up to 12 weeks and provide fewer outcome measures compared to other trials. This inconsistency poses challenges for robust statistical analysis. Additionally, the follow-up periods in the included studies are relatively short, restricting the ability to evaluate long-term disease management, which is crucial given the chronic nature of PN. To address these limitations, future research should prioritize longer follow-up periods to better assess the long-term efficacy and continued effectiveness of Nemolizumab.

Another limitation is the relatively small sample sizes in the included studies. Although placebo-controlled comparisons are valuable, the lack of direct head-to-head comparisons with other biologics limits the ability to position Nemolizumab precisely within the broader therapeutic landscape. Furthermore, all three included trials were industry-sponsored, raising concerns about potential selective reporting, where positive outcomes may be emphasized, and negative or inconclusive results could be underreported or unpublished. Due to the limited number of studies, a formal assessment of publication bias, such as funnel plot analysis or a statistical test, was not performed. Such analyses are typically unreliable with a small sample size, as they lack the statistical power necessary to detect bias.^24^

Notwithstanding these challenges, this systematic review and meta-analysis offers a comprehensive overview of the most robust evidence regarding the use of Nemolizumab for PN treatment.

## Conclusion

The meta-analysis provides strong evidence for the efficacy of Nemolizumab in treating PN, demonstrating significant improvements in pruritus reduction, IGA success rates, and PAS > 75%. Additionally, the safety profile of Nemolizumab was favorable, with no significant difference in the incidence of adverse events or serious adverse events compared to placebo. These findings support the use of Nemolizumab as an effective and safe treatment option for PN.

## Financial support

None declared.

## Authors’ contributions

Ana Carolina Putini Vieira: The study concept and design; critical review of literature; data collection, analysis and interpretation; preparation and writing of the manuscript; final approval of the final version of the manuscript.

Maria Antônia Costa Cruz Akabane: Data collection, analysis and interpretation; statistical analysis; preparation and writing of the manuscript; final approval of the final version of the manuscript.

Gianna Carolinne Graff Caletti: Critical review of literature; final approval of the final version of the manuscript.

Kélen Klein Heffel: Data collection, analysis and interpretation; final approval of the final version of the manuscript.

Amanda Tauana Oliveira e Silva: The study concept and design; preparation and writing of the manuscript; effective participation in research orientation; manuscript critical review; final approval of the final version of the manuscript.

## Research data availability

The entire dataset supporting the results of this study was published in this article.

## Conflicts of interest

The authors have no conflicts of interest that are directly relevant to the content of this article.
