# Supplementary material for: Efficacy and safety of nemolizumab in prurigo nodularis: a systematic review and meta-analysis of randomized controlled trials
Source: An Bras Dermatol. 2025 Oct 18;100(6):501210. doi: 10.1016/j.abd.2025.501210 (PMC12555766; doi:10.1016/j.abd.2025.501210)
Supplement: Supplementary file 1 [file mmc1.docx]

**ABD-D-25-00047**

**Supplementary Material**

**Supplementary Figure 1 Percent change from baseline in the Peak Pruritus Numerical Rating Scale at week-4.** Three studies were included in this analysis. The studies by Ständer 2020, Kwatra 2023 and Ständer 2024 provided data for this outcome. When pooling the results, Nemolizumab demonstrated a significant percent change from baseline in the Peak Pruritus Numerical Rating Scale at week-4 (MD = -32.04; 95% CI: -38.47, -25.62; p ≤ 0.001; I^2^ = 0%), favoring the intervention.

**Supplementary Figure 2 Percent change from baseline in the Peak Pruritus Numerical Rating Scale at week-12.** Three studies were included in this analysis. The studies by Ständer 2020, Kwatra 2023 and Ständer 2024 provided data for this outcome. When pooling the results, no significant difference was observed between the groups (MD = -347.34; 95% CI: -1039.71, 345.04; p = 0.325; I^2^ = 0%).

**Supplementary Figure 3 Discontinuation due to Adverse Events.** Three studies were included in this analysis. The studies by Ständer 2020, Kwatra 2023 and Ständer 2024 provided data for this outcome. When pooling the results, no significant difference was observed between the groups (RR = 1.09; 95% CI: 0.46, 2.54; p = 0.849; I^2^ = 0%).

**Supplementary Figure 4 Injection-related reactions.** Three studies were included in this analysis. The studies by Ständer 2020, Kwatra 2023 and Ständer 2024 provided data for this outcome. When pooling the results, no significant difference was observed between the groups (RR = 2.31; 95% CI: 0.38, 14.02; p = 0.364; I^2^ = 0%).

**Supplementary Figure 5 Peripheral edema.** Three studies were included in this analysis. The studies by Ständer 2020, Kwatra 2023 and Ständer 2024 provided data for this outcome. When pooling the results, no significant difference was observed between the groups (RR = 1.54; 95% CI: 0.40, 6.00; p = 0.530; I^2^ = 0%).

**Supplementary Figure 6 Urticaria.** Three studies were included in this analysis. The studies by Ständer 2020, Kwatra 2023 and Ständer 2024 provided data for this outcome. When pooling the results, no significant difference was observed between the groups (RR = 1.94; 95% CI: 0.31, 12.19; p = 0.479; I^2^ = 0%).

**Supplementary Figure 7 Contact dermatitis.** Three studies were included in this analysis. The studies by Ständer 2020, Kwatra 2023 and Ständer 2024 provided data for this outcome. When pooling the results, no significant difference was observed between the groups (RR = 3.62; 95% CI: 0.64, 20.35; p = 0.145; I^2^ = 0%).

**Supplementary Figure 8 Eczema.** Three studies were included in this analysis. The studies by Ständer 2020, Kwatra 2023 and Ständer 2024 provided data for this outcome. When pooling the results, no significant difference was observed between the groups (RR = 1.75; 95% CI: 0.39, 7.78; p = 0.465; I^2^ = 28%).

**Supplementary Figure 9 Alopecia.** Three studies were included in this analysis. The studies by Ständer 2020, Kwatra 2023 and Ständer 2024 provided data for this outcome. When pooling the results, no significant difference was observed between the groups (RR = 0.71; 95% CI: 0.14, 3.57; p = 0.681; I^2^ = 0%).

**Supplementary Figure 10 Rash.** Three studies were included in this analysis. The studies by Ständer 2020, Kwatra 2023 and Ständer 2024 provided data for this outcome. When pooling the results, no significant difference was observed between the groups (RR = 1.30; 95% CI: 0.28, 6.10; p = 0.738; I^2^ = 0%).

**Supplementary Figure 11 Rosacea.** Three studies were included in this analysis. The studies by Ständer 2020, Kwatra 2023 and Ständer 2024 provided data for this outcome. When pooling the results, no significant difference was observed between the groups (RR = 0.45; 95% CI: 0.07, 2.81; p = 0.390; I^2^ = 0%).

**Supplementary Figure 12 Dry skin.** Three studies were included in this analysis. The studies by Ständer 2020, Kwatra 2023 and Ständer 2024 provided data for this outcome. When pooling the results, no significant difference was observed between the groups (RR = 0.60; 95% CI: 0.12, 3.08; p = 0.539; I^2^ = 1%).

**Supplementary Figure 13 Neurodermatitis.** Three studies were included in this analysis. The studies by Ständer 2020, Kwatra 2023 and Ständer 2024 provided data for this outcome. When pooling the results, Nemolizumab showed a significant reduction in neurodermatitis compared to placebo (RR = 0.50; 95% CI: 0.32, 0.80; p = 0.004; I^2^ = 11%).

**Supplementary Figure 14 Cardiac disorders.** Three studies were included in this analysis. The studies by Ständer 2020, Kwatra 2023 and Ständer 2024 provided data for this outcome. When pooling the results, no significant difference was observed between the groups (RR = 0.56; 95% CI: 0.14, 2.20; p = 0.411; I^2^ = 0%).

**Supplementary Figure 15 Gastrointestinal disorders.** Three studies were included in this analysis. The studies by Ständer 2020, Kwatra 2023 and Ständer 2024 provided data for this outcome. When pooling the results, no significant difference was observed between the groups (RR = 0.89; 95% CI: 0.48, 1.65; p = 0.707; I^2^ = 19%).

**Supplementary Figure 16 Skin and subcutaneous tissue disorders.** Three studies were included in this analysis. The studies by Ständer 2020, Kwatra 2023 and Ständer 2024 provided data for this outcome. When pooling the results, no significant difference was observed between the groups (RR = 1.03; 95% CI: 0.79, 1.35; p = 0.89; I^2^ = 0%).

**Supplementary Figure 17 Infections and infestations.** Three studies were included in this analysis. The studies by Ständer 2020, Kwatra 2023 and Ständer 2024 provided data for this outcome. When pooling the results, no significant difference was observed between the groups (RR = 0.98; 95% CI: 0.66, 1.44; p = 0.899; I^2^ = 0%).

**Supplementary Figure 18 Musculoskeletal and connective-tissue disorders.** Three studies were included in this analysis. The studies by Ständer 2020, Kwatra 2023 and Ständer 2024 provided data for this outcome. When pooling the results, no significant difference was observed between the groups (RR = 1.48; 95% CI: 0.89, 2.46; p = 0.132; I^2^ = 0%).

**Supplementary Figure 19 Nervous system disorders.** Three studies were included in this analysis. The studies by Ständer 2020, Kwatra 2023 and Ständer 2024 provided data for this outcome. When pooling the results, no significant difference was observed between the groups (RR = 1.10; 95% CI: 0.65, 1.84; p = 0.730; I^2^ = 0%).

**Supplementary Figure 20 Respiratory, thoracic and mediastinal disorders.** Three studies were included in this analysis. The studies by Ständer 2020, Kwatra 2023 and Ständer 2024 provided data for this outcome. When pooling the results, no significant difference was observed between the groups (RR = 0.93; 95% CI: 0.29, 3.01; p = 0.3839; I^2^ = 0%).
